# Autoimmune Encephalitis and Related Syndromes

**DOI:** 10.3390/jcm12113832

**Published:** 2023-06-03

**Authors:** Francesc Graus

**Affiliations:** Neuroimmunology Program, Institut d’Investigacions Biomèdiques August Pi i Sunyer (IDIBAPS), Casanova, 143, Floor 3rd, 08036 Barcelona, Spain; francesc.graus@idibaps.org; Tel.: +34-932271738

The field of autoimmune neurology has greatly expanded in the last decade. At the turn of the century, autoimmune disorders of the central nervous system included multiple sclerosis, a disease that mainly affects the white matter, and paraneoplastic neurological syndromes (PNSs), which encompasses a group of disorders that frequently associates with cancer and cause severe, usually irreversible, neuronal cell loss. In both disorders, the pathogenesis is considered to be T-cell-mediated.

The discovery of neural (neuronal and glial) antibodies against synaptic receptors and other surface antigens has altered the landscape of autoimmune neurological diseases of the central nervous system forever. In the field of demyelinating syndromes, neuromyelitis optica spectrum disorders (NMOSD) associated with aquaporin 4 antibodies and myelin oligodendrocyte glycoprotein (MOG) antibody-associated disease (MOGAD) have been recognized as antibody-mediated diseases, with a clinical course and response to immunotherapy different from multiple sclerosis. PNSs as a cause of autoimmune damage have been surpassed by the characterization of autoimmune encephalitis caused by antibodies against synaptic receptors and other surface proteins, being the most common anti-N-methyl-D-aspartate (NMDA) receptor encephalitis in children and young women, and leucine-rich glioma-inactivated 1 (LGI1) encephalitis in elderly men [1]. At present, autoimmune encephalitis has become the leading differential diagnosis in patients with subacute altered mental status, psychiatric alterations, seizures, or cognitive impairment. Testing for neuronal antibodies has significantly increased, as their presence confirms the diagnosis and is helpful for identifying different co-morbidities, such as the presence of an underlying tumor, and can assist to define the prognosis.

Over the last three years, the journal *Clinical Medicine* has published several articles that present new and relevant aspects of autoimmune encephalitis, PNS, and related autoimmune disorders. This editorial highlights four autoimmune disorders based on their relevance in the diagnosis and management.

PNSs are rare and occur in <1% of cancer patients; however, due to their severity, they have a significant impact on the patient’s quality of life. Virtually all types of tumors have been associated with PNS, but lung cancer, especially small cell cancer (SCLC), is characterized by a highest incidence. In a prospective study of 264 patients with SCLC, 24 (9%) developed a PNS, mainly Lambert–Eaton myasthenic syndrome, sensory neuronopathy, and limbic encephalitis [2]. Epidemiological studies to assess the incidence and prevalence of PNSs were lacking until 2020, when three studies addressed this issue [3,4,5]. In one of them, published in this journal, Lorusso and colleagues evaluated the prevalence of PNSs in the region of Lombardy, Italy, in the period of 1998 to 2003. Only PNSs admitted to hospital were identified. The authors found a prevalence of 5.92 cases per 100,000 inhabitants [5]. This prevalence was slightly higher than the 4 cases per 100,000 inhabitants found in the epidemiologic study done in the region of Friuli-Venezia Giulia region in Italy and the 5.4 cases per 100,000 inhabitants in the population-based study in Olmsted County, Minnesota, USA [3,6]. The higher prevalence in the study of Lorusso and colleagues was justified by the high prevalence of cancer in the region of Lombardy, which is one of the highest in Italy [5].

Although not reported in Lorusso’s study, other epidemiological studies have consistently observed an increase in the annual incidence of PNSs over the last years [3,4,6]. For example, in the population-based study in Olmsted County, Minnesota, USA, PNS incidence increased from 0.4 per 100,000 person-years in the 1987–2002 period, to 0.8 per 100,000 person-years in the 2003–2018 period [6]. The increased incidence likely reflects a greater awareness of PNSs by neurologists and a wider accessibility to onconeural antibody testing. Another plausible explanation for the increase in the frequency of PNSs is the increasing use of monoclonal antibodies against immune checkpoint molecules that normally inhibit T-cell activation in the treatment of cancer. These immune checkpoint inhibitors, particularly those against programmed cell death 1 (PD-1) and its ligand (PDL1), have been associated with an increased risk for developing PNS, which is explained by the effect of the immune checkpoint inhibitors in the activation of the immune system [7]. In an experimental model, mice expressing a neo-self-antigen in Purkinje cells and an implanted tumor received antigen-specific CD4 and CD8 T-cells with or without an immune checkpoint inhibitor. The animals treated with the immune checkpoint inhibitor developed prominent neurological deficits and Purkinje cell loss caused by CD8+ cytotoxic T cells specifically directed against the neo-self-antigen [8].

A critical step in the management of PNSs is the prompt identification of the underlying tumor which, in many instances, is not present at the time of PNS diagnosis. Full-body computed tomography (CT) or fluorodeoxyglucose positron emission tomography combined with CT (FDG-PET/CT) are preferred screenings for a vast majority of tumors. Other imaging procedures are indicated for particular tumors, such as mammography or breast MRI in suspected breast cancer, testicular ultrasound for suspected testicular cancer, and transvaginal ultrasound for ovarian teratoma.

FDG-PET/CT is increasingly used as the first screening tool in some centers. In the single-center retrospective study of Opalinska and colleagues, 15 patients with suspected PNS were evaluated via FDG PET/CT, and a tumor was confirmed in 8 (53%) [9]. The frequency was lower than the 87% and 89% obtained in two meta-analyses [10,11]. The low sensitivity in Opalinska’s study may be explained by the inclusion of patients with diagnoses (myasthenia gravis, primary angiitis of the central nervous system) which are not classical or high-risk PNSs. The study also included patients who were suspected to have a PNS on the basis of a positive Yo antibody. In many centers, Yo antibodies are determined only using commercial line blots, and if the result is not confirmed through immunohistochemistry on rodent cerebellum, the specificity for PNS is very low. In fact, there is no indication to search for an underlying cancer on the basis of a positive result for Yo antibodies only evaluated using commercial line blots [12]. An issue not addressed in Opalinska’s study is the frequency of false positive results, leading to unnecessary diagnostic procedures that may occur in up to 20% of FDG PET/CT studies [13].

Another unresolved issue is how to proceed for patients with suspected PNS when the first search for cancer is negative. In the report of the EFNS task force of 2011, experts recommended repeating screening every 6 months up to 4 years [14]. However, in a more recent consensus paper, this period was reduced to 2 years as the vast majority of tumors in patients with PNS are diagnosed <2 years after PNS onset [15]. Patients with classical PNS should be re-evaluated throughout at least 2 years, unless they present antibodies that do not associate with cancer (for example limbic encephalitis and LGI1 antibodies). This recommendation also applies for any neurologic syndrome of unknown cause associated with onconeural antibodies.

Autoimmune brainstem encephalitis is caused by different etiologies and in some instances, the clinical and imaging findings are helpful to indicate the diagnosis. Autoimmune brainstem encephalitis can be classified as idiopathic, either primary or secondary to systemic autoimmune diseases, and paraneoplastic. Zoghaib and colleagues reported a patient with a brainstem syndrome associated with MOG antibodies in the journal *Clinical Medicine*, and reviewed other causes of autoimmune brainstem encephalitis [16]. Among primary idiopathic brainstem encephalitis, the differential diagnosis between multiple sclerosis, NMOSD, and MOGAD may sometimes be difficult. In a retrospective observational study, brainstem/cerebellar involvement was reviewed in patients with MOGAD, NMOSD, and multiple sclerosis. Brainstem manifestations occurred in 62/185 (34%) MOGAD patients. Isolated attacks were less frequent in MOGAD (23%) than multiple sclerosis (73%) and NMOSD (47%). In the brain MRI, diffuse middle cerebellar peduncle FAIR/T2-weighted lesions were also more common in MOGAD (46%) over MS (10%) and NMOSD (10%). Conversely, cerebrospinal fluid (CSF) oligoclonal bands were present in 82% of patients with MS, but only in <20% of those with MOGAD or NMOSD [17].

In their review, Zoghaib and colleagues include leucine zipper 4 (LUZP4) antibodies in the list of onconeural antibodies that associate with paraneoplastic brainstem encephalitis [16]. However, LUZP4 antibodies are frequently associated (~80% of patients) with Ma2, and particularly kelch-like protein 11 (KLHL11) antibodies in patients with brainstem encephalitis and testicular seminomas, indicating that in most cases, LUZP4 antibodies do not add diagnostic value to the detection of Ma2 or KLHL11 antibodies [18]. Future studies are needed to clarify the real value of LUZP4 antibodies in the diagnosis of paraneoplastic brainstem encephalitis.

The first-line treatment of autoimmune encephalitis and PNSs includes intravenous methylprednisolone (1 g/d for 3 to 5 days) or intravenous immunoglobulins (2 g divided in 5 days) and less frequently oral prednisone (1 mg/Kg/day). These treatments are usually not very effective in PNS associated with onconeural antibodies, as they likely cause neuronal death mediated by T-cell cytotoxicity. In patients with autoimmune encephalitis triggered by antibodies against synaptic receptors and other surface antigens, a partial improvement or complete recovery occur in at least 75% of the cases. The role of plasma exchange as an alternative or add-on therapy is a matter of debate. Rössling and Prüss addressed this issue by a systematic review of the literature using the PRISMA (Preferred Reporting Items for Systematic Reviews and Meta-Analyses) guidelines and screening the articles independently for their respective eligibility [19]. They identified four retrospective and two prospective studies that evaluated the effect of plasma exchange or immunoadsorption in patients with autoimmune encephalitis and PNS. The apheresis was used as an add-on therapy or in patients with suboptimal response to first-line therapies in most of the studies. Although the design of the analyzed studies prevented drawing firm conclusions, two messages are important: first, as expected, apheresis did not work on neurologic syndromes associated with antibodies against intracellular antigens; and second, in autoimmune encephalitis, a better outcome was strongly associated with an early start of the treatment [19].

Improvement in the outcome of autoimmune encephalitis will require the evaluation of new drugs tested in rigorous clinical trials. At present, Inebilizumab, a B-cell-depleting anti-CD19 humanized antibody that can be administered intravenously with good CSF penetration, will be tested in a phase 2B randomized, double-blind, placebo-controlled trial (the ExTINGUISH trial) to assess the safety and efficacy in patients with anti-NMDAR encephalitis. All patients will receive standard first-line immunotherapies prior to randomization. Primary outcomes will be ascertained at 16 weeks using the change in modified Rankin scale. In a similar trial, rozanolixizumab, an inhibitor of neonatal Fc receptor that favors the degradation of immunoglobulins, will be tested in a randomized, double-blind, placebo-controlled, phase 2 study of patients with anti-LGI1 encephalitis, to assess the safety and efficacy measured by seizure freedom, change in cognitive function, use of rescue medication, and the onset of seizure freedom.

Better treatments for autoimmune encephalitis and PNSs are urgently needed. For the design of clinical trials to improve the outcome, it is necessary to increase our knowledge on the frequency, clinical features, and management of these disorders, as accomplished in these selected manuscripts published in the journal *Clinical Medicine*.
